# Dysbiotic microbiome variation in colorectal cancer patients is linked to lifestyles and metabolic diseases

**DOI:** 10.1186/s12866-023-02771-7

**Published:** 2023-01-28

**Authors:** Tung Hoang, Minjung Kim, Ji Won Park, Seung-Yong Jeong, Jeeyoo Lee, Aesun Shin

**Affiliations:** 1 Department of Preventive Medicine, Seoul National University College of Medicine, Seoul, 03080 South Korea; 2 Integrated Major in Innovative Medical Science, Seoul National University College of Medicine, Seoul, 03080 South Korea; 3 Department of Surgery, Seoul National University College of Medicine, Seoul, 03080 South Korea; 4 Cancer Research Institute, Seoul National University, Seoul, 03080 South Korea

**Keywords:** Anna Karenina principle, Dysbiosis, Colorectal cancer, Lifestyle, Metabolic disease, Diet

## Abstract

**Background:**

Differences in the composition and diversity of the gut microbial communities among individuals are influenced by environmental factors. However, there is limited research on factors affecting microbiome variation in colorectal cancer patients, who display lower inter-individual variations than that of healthy individuals. In this study, we examined the association between modifiable factors and the microbiome variation in colorectal cancer patients.

**Methods:**

A total of 331 colorectal cancer patients who underwent resection surgery at the Department of Surgery, Seoul National University Hospital between October 2017 and August 2019 were included. Fecal samples from colorectal cancer patients were collected prior to the surgery. Variations in the gut microbiome among patients with different lifestyles and metabolic diseases were examined through the network analysis of inter-connected microbial abundance, the assessment of the Anna Karenina principle effect for microbial stochasticity, and the identification of the enriched bacteria using linear discrimination analysis effect size. Associations of dietary diversity with microbiome variation were investigated using the Procrustes analysis.

**Results:**

We found stronger network connectivity of microbial communities in non-smokers, non-drinkers, obese individuals, hypertensive subjects, and individuals without diabetes than in their counterparts. The Anna Karenina principle effect was found for history of smoking, alcohol consumption, and diabetes (with significantly greater intra-sample similarity index), whereas obesity and hypertension showed the anti-Anna Karenina principle effect (with significantly lower intra-sample similarity index). We found certain bacterial taxa to be significantly enriched in patients of different categories of lifestyles and metabolic diseases using linear discrimination analysis. Diversity of food and nutrient intake did not shape the microbial diversity between individuals (p_Procrustes_>0.05).

**Conclusions:**

Our findings suggested an immune dysregulation and a reduced ability of the host and its microbiome in regulating the community composition. History of smoking, alcohol consumption, and diabetes were shown to affect partial individuals in shifting new microbial communities, whereas obesity and history of hypertension appeared to affect majority of individuals and shifted to drastic reductions in microbial compositions. Understanding the contribution of modifiable factors to microbial stochasticity may provide insights into how the microbiome regulates effects of these factors on the health outcomes of colorectal cancer patients.

**Supplementary Information:**

The online version contains supplementary material available at 10.1186/s12866-023-02771-7.

## Introduction

Given the complex and dynamic nature of the microbial community in the gastrointestinal tract, the gut microbiome can actively interact with immune cells through the intestinal mucosal surface, and provide the host with colonization resistance against foreign microbes [1–3]. When the bacterial homeostasis is disrupted, there is an imbalance between the commensal and pathogenic bacteria in the gut that can lead to the formation of inflammatory biomarkers and stimulate the carcinogenesis process [1, 4]. This condition is called dysbiosis, which is determined to involve several physiological processes, such as inflammation, pathogenic bacteria, genotoxins, oxidative stress, metabolites, and biofilm [5].

To date, several researchers have proposed using an Anna Karenina principle (AKP) effect to describe the increase of stochastic transitions from stable to unstable states in the gut microbiome [6]. Regarding microbiome-associated factors, the AKP has been called “all healthy microbiomes are similar; each dysbiotic microbiome is dysbiotic in its own way” [6]. The AKP effect, therefore, indicates the increase of microbiome heterogeneity or stochasticity related to dysbiosis due to abnormal conditions and the high personalization of factor-associated microbial communities [7].

Differences in the composition and diversity of the gut microbial communities among individuals are contributed by environmental factors [8, 9]. Lifestyle, diet, and chronic disease have been generally described to affect specific components of the gut microbiome [10]. Under some conditions of immune system dysfunctions, there could be a rise of stochasticity and the dysbiotic communities much more veried from person to person [7]. However, the composition of the microbiome community in colorectal cancer (CRC) patients differs from the core microbiome and diversity levels of healthy individuals [11, 12]. More research is required to identify how modifiable factors affect the microbial instability in CRC patients and thus understand how the gut microbiome may regulate the effect of modifiable factors on the health outcomes of CRC patients.

In CRC patients, metabolic syndrome and lifestyle behaviors have been shown to contribute to the patient's prognosis [13, 14]. In addition, dietary factors can modify the gut microbial community via energy harvesting and several diet-derived metabolites, such as short-chain fatty acids (SCFAs) [15].^11^ Although several data-driven approaches are available for the determination of a person’s dietary patterns [16], individuals may differ not only in the specific food items they eat but also in their dietary diversity [17]. However, such a tree-based approach has not been applied to the habitual diets of the Korean population.

In this study, we first created the hierarchical tree of foods to reflect dietary diversity and investigated its associations with lifestyle factors and metabolic diseases. Then, we examined the association of lifestyle factors, metabolic disease, and dietary intake with the gut microbiome variation. We hypothesized that CRC participants with unhealthy lifestyles, such as smoking and alcohol consumption, and metabolic diseases may associate with a higher level of microbiome stochasticity.

## Methods

### Healthy lifestyles and non-metabolic diseases in the majority of study participants

The study was designed as a cross-sectional study and included study subjects who were diagnosed with CRC and underwent resection surgery between October 2017 and August 2019 in the Department of Surgery, Seoul National University Hospital, Seoul, Korea. Among the selected CRC patients, a total of 331 patients were included in this study after excluding those who could not be analyzed due to the absence or small amount of fecal sample prior to the surgery. Of these, 115 patients provided their dietary information.

Demographic characteristics, lifestyle behaviors, and metabolic diseases of CRC patients are shown in Table 1. The mean age of study participants was 61.9 years, with 14.8% of patients being early-onset (age ≤50 years old) cases (*n=*49). Of the included patients, 19.6% were ever smokers (*n=*65), 38.1% ever consumed alcohol (*n=*126), 40.5% were obese (BMI≥25.0 kg/m^2^) (*n=*134), 39.3% had hypertension (*n=*130), and and 21.1% had diabetes (*n=*70). Patient demographics, family history of CRC, neoadjuvant therapy, lifestyles, and metabolic diseases did not differ between those with and without dietary information (*p>*0.05).

**Table 1:** Characteristics of study participants

**Factor**	**Total (*N=*331)**	**With dietary data (*N=*115)**	**Without dietary data (*N=*216)**	***P*-value**
**Age (mean ± sd, years)**	61.9 ± 11.0	60.8 ± 11.8	62.4 ± 10.5	0.21
≤50	49 (14.8)	23 (20.0)	26 (12.0)	0.23
50-≤60	94 (28.4)	31 (27.0)	63 (29.2)	
60-≤70	118 (35.6)	36 (31.3)	82 (38.0)	
>70	70 (21.1)	25 (21.7)	45 (20.8)	
**Sex**				
Female	122 (36.9)	41 (35.7)	81 (37.5)	0.83
Male	209 (63.1)	74 (64.3)	135 (62.5)	
**Family history of CRC**				
No	291 (87.9)	105 (91.3)	186 (86.1)	0.23
Yes	40 (12.1)	10 (8.7)	30 (13.9)	
**Neoadjuvant therapy**				
No	290 (87.6)	104 (90.4)	186 (86.1)	0.34
Chemotherapy and/or radiotherapy	41 (12.4)	11 (9.6)	30 (13.9)	
**Smoking status**				
Never	266 (80.4)	92 (80.0)	174 (80.6)	>0.99
Ever	65 (19.6)	23 (20.0)	42 (19.4)	
**Alcohol consumption**				
Never	205 (61.9)	74 (64.3)	131 (60.6)	0.59
Ever	126 (38.1)	41 (35.7)	85 (39.4)	
**BMI (mean ± sd, kg/m**^**2**^**)**	24.4 ± 3.2	24.6 ± 3.3	24.3 ± 3.2	0.44
Normal (<25.0)	197 (59.5)	38 (33.0)	136 (63.0)	0.10
Obesity (≥25.0)	134 (40.5)	77 (67.0)	80 (37.0)	
**Hypertension**				
No	201 (60.7)	75 (65.2)	126 (58.3)	0.27
Yes	130 (39.3)	40 (34.8)	90 (41.7)	
**Diabetes**				
No	261 (78.9)	94 (81.7)	167 (77.3)	0.43
Yes	70 (21.1)	21 (18.3)	49 (22.7)	

Data are presented as mean ± standard deviation for continuous variables and counts (percentages) for categorical variables

*P*-values are calculated from a t-test for continuous variables and a Chi-square test for categorical variables

*CRC* Colorectal cancer, *BMI* Body mass index

### Collection of lifestyles, dietary habits, and clinical data

On the study enrollment, we obtained patient information on age, sex, family history of CRC, neoadjuvant therapy, tobacco smoking and alcohol consumption experiences, and history of hypertension and diabetes. Additionally, the height and weight of the patients were measured and used to calculate the body mass index (BMI). After surgery, the disease stage was further assessed following the American Joint Committee on Cancer (AJCC) criteria. The average amount of diet consumption during the preceding year was assessed using validated a semi-quantitative food frequency questionnaire (SQFFQ), which was developed by the Korea Centers for Disease Control and Prevention [18]. By using the Computer-Aided Nutritional Analysis Program (CAN-Pro) 4.0 (Korean Nutrition Information Center, Seoul, Korea), we estimated the weight intake of macro- and micronutrients from 663 food subitems, which were generated from 106 food items in the SQFFQ. From this, 35 food groups were generated, which has been applied in previous studies [19]. The CAN-Pro software further identified the higher level with 17 food groups [20] and we determined the highest level with the plant-based foods, animal-based foods, beverages, and condiments. Details for the components of the tree-based diet are available in (Additional File 1: eTable1).

### Fecal sample collection and 16S rRNA sequencing process

In this study, participants received a kit (DNeasyPowerSoil Kit, Qiagen, Hilden, Germany) and collected a single stool sample according to the kit instructions. The sample was collected before the operation date, and fecal microbiota was analyzed using 16S rRNA gene amplicon sequencing with V3-V4 primers. The first polymerase chain reaction (PCR) product was purified with AMPure beads (Agencourt Bioscience, Beverly, MA). Following purification, 2μl of the first PCR product was PCR amplified for final library construction containing the index using NexteraXT Indexed Primer. The cycle conditions for the second PCR were the same as the first PCR except for 10 cycles. The final PCR product was purified with AMPure beads and then quantified using qPCR according to the qPCR Quantification Protocol Guide (KAPA Library Quantificatoin kits for IlluminaSequecing platforms) and qualified using the TapeStation D1000 ScreenTape (Agilent Technologies, Waldbronn, Germany). The paired-end (2×300 bp) sequencing was performed by the Macrogen using the MiSeq™ platform (Illumina, San Diego, USA). The number of operational taxonomic units was identified by utilizing the preprocessed reads of samples and clustering the sequences from samples using a 97% sequence identity cut-off.

### Statistical analysis

#### Lifestyle factors, metabolic diseases, and microbial variation

The interaction of microbiome relative abundance was visualized using a network analysis approach. Given the compositional and zero-inflated properties of the microbiome data, numerous correlation-focused approaches have been developed to overcome the difficulty of inferring dependencies in microbial data, such as CCREPE, SparCC, CCLasso, and REBACCA [21, 22]. However, these methods may be limited in reflecting indirect relationships and causing spurious associations from the creation of pseudo-counts [21, 22]. Ha et al. introduced a COpositional Zero-Inflated Network Estimation (COZINE) to address this challenge by generating a binary incidence matrix and a compositional abundance matrix in which the centered log-ratio transformation can be applied for non-zero counts only [21, 22]. In this study, the network structure was constructed for each group of smoking status, alcohol consumption, BMI, and underlying diseases using the COZINE method (R packages ‘COZINE’ and ‘HurdleNormal’) [21, 22].

For the estimation of the AKP effect on the increase of microbial stochasticity associated with dysbiosis due to the external factor, Ning et al. recently developed a framework to assess and present the ecological stochasticity as a single index, which is called normalized stochasticity ratio [23]. Borrowing the Ružička similarity concept from this framework, we calculated the intra-sample similarity (C) index to reflect the similarity in microbiome composition of individuals in each group according to exposure status [7]. In general, if the C-index of the exposed group was higher than that of the non-exposed group, it implied presence of the AKP effect according to that exposed factor and the higher stochasticity or heterogeneity in the exposed group [7]. The C-indexes of groups were then compared using a Wilcoxon test, with the level of significance defined as *p<*0.05.

Furthermore, we conducted the linear discrimination analysis effect size (LEfSe) to identify bacteria that are phylogenetically abundant in each group of lifestyle factors and metabolic diseases.

#### Dietary diversity and microbial variation

Our previous study identified several dietary factors that were correlated with the relative abundance of several taxon [24], however, whether the overall diversity of source food intake reflecting differences of microbiome composition among CRC patients remained unclear. Given an estimate of almost 10% of dietary energy was obtained due to microbial fermentation and volatile fatty acid production [25], we used a tree-based approach to assess the dietary diversity of energy intake and its components in associations with microbiome variations. The assessment of dietary diversity included considerations of the consumption of energy intake (kcal/day), plant/animal protein (g/day), plant/animal fat (g/day), carbohydrate (g/day), and fiber (g/day). Based on the tree-based structure of dietary intake, we calculated Chao1, Shannon, and Simpson indices for within-subject (alpha)-diversity, which reflected the overall diversity of food source consumption within each patient. In addition, to capture the variation from patient to patient in terms of dietary intake composition, we calculated unweighted and weighted UniFrac distances for between-subject (beta)-diversity of dietary intake (R packages ‘vegan’ and ‘GuniFrac’).

To test for the association of dietary diversity with microbiome variation across subjects, we performed the Procrustes analysis to compare the shapes of two beta-diversity matrixes by translating, rotating, and uniformly scaling the matrixes (R package ‘ape’).

#### Dietary diversity in associations with lifestyle factors and metabolic diseases

To explore whether the AKP or anti-AKP effect of lifestyle factors and metabolic diseases might be attributed by the dietary diversity,we examined the difference of dietary diversity indices according to lifestyle factors and metabolic diseases. Thus, we applied the generalized linear model to investigate the association of alpha-diversity and the permutational multivariate analysis of variance (PERMANOVA) test to investigate the association of beta-diversity of diet consumption with lifestyle factors and metabolic diseases. Significant differences in the alpha- and beta-diversity according to lifestyle factors and metabolic diseases were visualized as box plot and principal coordinate analysis plots.

The LEfSe analysis was performed in the Galaxy web application (https://huttenhower.sph.harvard.edu/galaxy/) and other statistical analyses were performed in R 3.6.0.

## Results

### Lifestyle factors and metabolic diseases and microbial variation

#### Network structure for the partial correlation of the gut microbiome in CRC

**Fig. 1:**
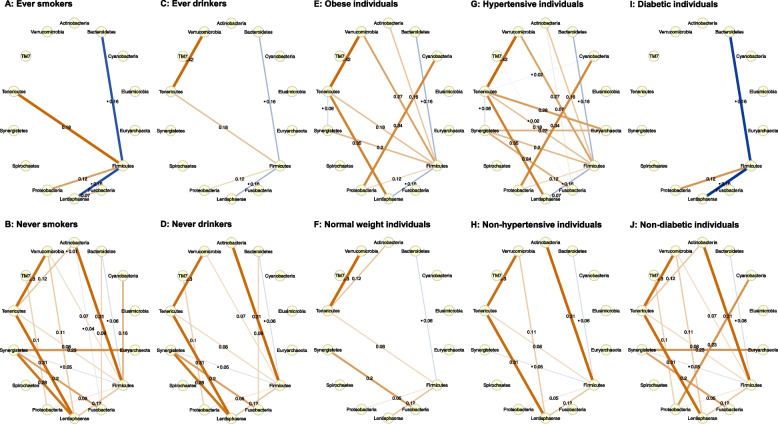
COpositional Zero-Inflated Network Estimation (COZINE) identifies network structure of phylum abundance in **(A)** ever smoking, **(B)** never smoking, **(C)** ever drinking, **(D)** never drinking, **(E)** obese, **(F)** normal weight, **(G)** hypertensive, **(H)** non-hypertensive, **(I)** diabetic, and **(J)** non-diabetic individuals. Nodes represent the abundance of phyla, and edges represent the partial correlation coefficient between phyla. Brown lines show positive partial correlations, and blue lines show negative partial correlations. The thickness of the edges is proportional to their partial correlations

The Spearman partial correlation between phyla across different population groups is shown in Figs. 1A-1J. Significant non-zero edges were identified in the COZINE framework. The networks of phylum abundance in the exposed group of smoking status, alcohol consumption, or diabetes were relatively sparse compared to the non-exposed group, whereas the networks of phylum abundance in the obese or hypertensive group were relatively dense compared to the counterpart. In these groups of patients with more connected microbial communities (never smokers, never drinkers, obese, hypertensive, and non-diabetic individuals), there were strong abundance correlations between *Tenericutes* and *Verrucomicrobia* (*ρ=*0.30 to *ρ=*0.42), *Tenericutes* and *Lentisphaerae* (*ρ=*0.31 to *ρ=*0.35). Nevertheless, abundances between *Bacteroidetes* and *Firmicutes* phyla, which were the two most abundant phyla, were negatively correlated (*ρ=*-0.16 to *ρ=*-0.06). Of all communities, *Firmicutes* appeared to be the central phylum, which mostly connected with other phyla in the networks.

#### Assessment of the Anna Karenina principle effect

Table 2 presents the summary statistics of medians and interquartile ranges and *p*-values from the Wilcoxon test for the difference in intra-sample similarity (C) index. Accordingly, smoking, drinking, and diabetes exhibited the AKP effect (*p<*0.05 for the null hypothesis that C index in the exposed group is higher than in the non-exposed group), whereas obesity and hypertension exhibit the anti-AKP effect (*p<*0.05 for the null hypothesis of C index in the exposed group is less than in the non-exposed group). This suggested different responses among individuals who had history of tobacco smoking, alcohol consumption, and diabetes, that not all individuals showed shifts to new microbial compositions, as a result, resulted in an increase in beta-diversity. Under conditions of obesity and hypertension, all individuals were affected and showed shifts to new microbial compositions which were similar from person to person, which resulted in an excessive reduction in microbial compositions and lower beta-diversity compared to their counterparts.

**Table 2:** Intra-sample similarity index and Wilcoxon test for the detection of AKP effects of lifestyle factors and metabolic diseases

**Factor**	**Exposed (E) group **^**a**^	**Non-exposed (NE) group **^**a**^	***P*-value (E≠NE) **^**b**^	***P*-value (E>NE) **^**c**^	***P*-value (E<NE) **^**d**^
Smoking status	0.812 (0.695-0.877)	0.790 (0.690-0.870)	0.001	<0.001	>0.99
Alcohol consumption	0.815 (0.709-0.882)	0.784 (0.689-0.861)	<0.001	<0.001	>0.99
Obesity	0.778 (0.679-0.855)	0.807 (0.705-0.877)	<0.001	>0.99	<0.001
Hypertension	0.782 (0.678-0.861)	0.803 (0.705-0.874)	<0.001	>0.99	<0.001
Diabetes	0.804 (0.694-0.889)	0.790 (0.690-0.870)	<0.001	<0.001	>0.99

^a^Data are presented as median (interquartile range)

^b^*P*-value less than 0.05 indicates the presence of AKP or anti-AKP effect

^c^*P*-value less than 0.05 indicates AKP effect

^d^*P*-value less than 0.05 indicates anti-AKP effect

#### Differentially abundant bacteria

Bacteria that were highly enriched in individuals who have smoked on at least one occasion or have never smoked, who have consumed alcohol on at least one occasion or have never consumed alcohol, who are obese or are of normal weight, who have hypertension or do not have hypertension, and who are diabetic or non-diabetic are presented in (Additional File 1: eFigure1-5). The list of these taxa at different phylum, class, order, family, genus, and species levels is summarized in Table 3. Taxa related to class *Bacilli* were enriched in smokers, whereas taxa related to order *Desulfovibrionales* and *Synergistales* were enriched in non-smokers. Additionally, taxa related to family *Micrococcaceae*, *Enterococcaceae*, and *Enterobacteriaceae* were enriched in non-drinkers, and taxa related to class *Betaproteobacteria* was enriched in obese individuals. In terms of metabolic diseases, taxa related to phylum *Elusimicrobia* was enriched in individuals with hypertension and diabetes.

**Table 3:** Abundant bacteria identified by linear discriminant analysis effect size analysis in different statuses of lifestyles and metabolic diseases

**Factor**	**Phylum**	**Class**	**Order**	**Family**	**Genus**	**Species**
**Smoking status**						
Never	*Synergistetes*	*Deltaproteobacteria* *Synergistia*	*Desulfovibrionales* *Synergistales*	*Desulfovibrionaceae* *Synergistaceae*	*Bilophila* *Synergistes*	*Desulfovibrio D168* *Bilophila sp.* *Synergistes sp.*
Ever		*Bacilli*	*Lactobacillales*	*Streptococcaceae*	*Streptococcus* *Hafnia*	*Streptococcus sp.* *Hafnia alvei*
**Alcohol consumption**						
Never					*rc4_4*	*rc4_4 sp.*
Ever		*Gammaproteobacteria*	*Enterobacteriales*	*Micrococcaceae* *Enterobacteriaceae* *Enterococcaceae*	*Rothia* *Citrobacter* *Enterococcus*	*Citrobacter sp.* *Enterococcus sp.*
**Body mass index**						
<25.0 kg/m^2^			*SHA_98*		*Fusobacterium*	*Ruminococcus albus* *Peptostreptococcus anaerobius* *Streptococcus agalactiae* *Fusobacterium sp.*
≥25.0 kg/m^2^		*Betaproteobacteria*	*Burkholderiales*	*Lachnospiraceae* *Alcaligenaceae*	*Faecalibacterium* *Sutterella*	*Bacteroides coprophilus* *Clostridium baratii* *Faecalibacterium prausnitzi* *Blautia obeum* *Sutterella sp.*
**Hypertension**						
No					*Eubacterium*	*Eubacterium biforme*
Yes	*Elusimicrobia Synergisteles*	*Elusimicrobia* *Synergistia*	*Elusimicrobiales* *Synergistales*	*Elusimicrobiaceae*		*Desulfovibrio sp.*
**Diabetes**						
No	*Synergisteles*	*Synergistia*	*Synergistales*	*S24_7*	*Ruminococcus* *Barnesiella*	*Desulfovibrio D168* *Prevotella sp.* *Ruminococcus lactaris* *Barnesiella intesttinihominis*
Yes	*Elusimicrobia*	*Elusimicrobia*	*Lactobacillales* *Elusimicrobiales*	*Peptostreptococcaceae* *Streptococcaceae* *Elusimicrobiaceae*	*Veillonella* *Streptococcus*	*Desulfovibrio sp.* *Ruminococcus gnavus* *Veillonella dispar* *Streptococcus sp.*

### Dietary diversity and microbial variation

**Fig. 2:**
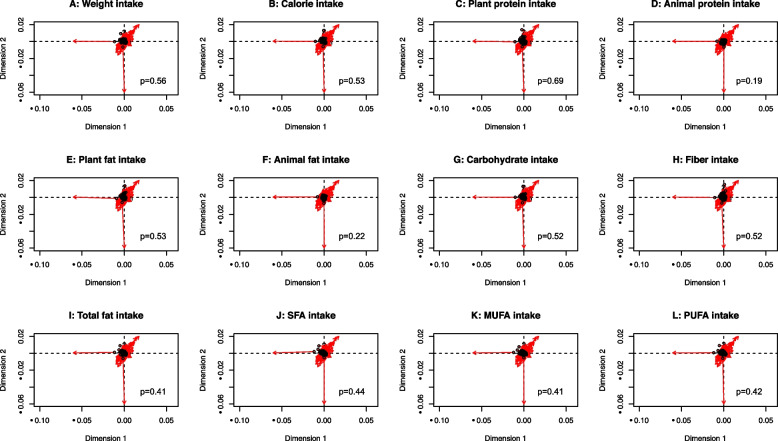
Procrustes analysis of tree-based food beta-diversity (unweighted UniFrac) of daily **(A)** weight consumption, **(B)** energy intake **(C)** plant protein, **(D)** animal protein, **(E)** plant fat, **(F)** animal fat, **(G)** carbohydrates, **(H)** fiber, **(I)** total fatty acids, **(J)** saturated fatty acids, **(K)** monounsaturated fatty acids, and **(L)** polyunsaturated fatty acids with microbiome composition beta-diversity (Aitchison’s distance). The plots show the rotation between the two ordinations necessary to make them match as closely as possible. Symbols (in black color) show the position of the samples in the first ordination (tree-based food), and arrows (in red color) point to their positions in the target ordination (microbiome composition)

**Fig. 3:**
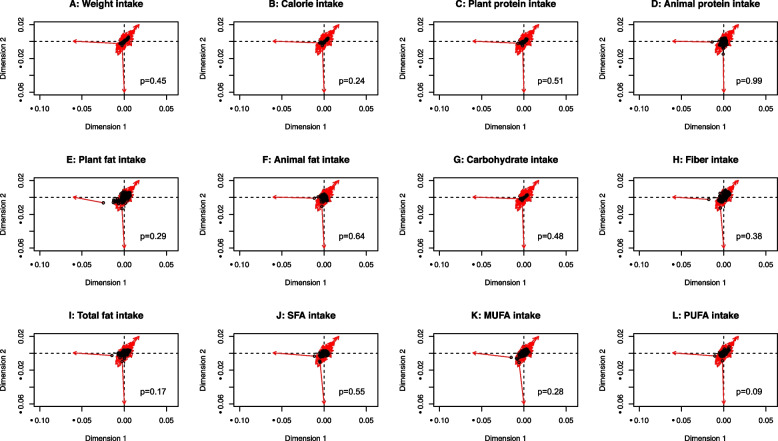
Procrustes analysis of tree-based food beta-diversity (weighted UniFrac) of daily **(A)** weight consumption, **(B)** energy intake **(C)** plant protein, **(D)** animal protein, **(E)** plant fat, **(F)** animal fat, **(G)** carbohydrates, **(H)** fiber, **(I)** total fatty acids, **(J)** saturated fatty acids, **(K)** monounsaturated fatty acids, and **(L)** polyunsaturated fatty acids with microbiome composition beta-diversity (Aitchison’s distance). The plots show the rotation between the two ordinations necessary to make them match as closely as possible. Symbols (in black color) show the position of the samples in the first ordination (tree-based food), and arrows (in red color) point to their positions in the target ordination (microbiome composition)

The rotation from the dietary beta-diversity matrix (for weight consumption, energy intake, plant protein, animal protein, plant fat, animal fat, carbohydrates, fiber, total fatty acids, saturated fatty acids, monounsaturated fatty acids (MUFAs), and polyunsaturated fatty acids (PUFAs)) into the microbiome diversity matrix was examined by the Procrustes analysis. We found that the food choice of an individual did not correspond with the microbiome composition of that individual when analyzed using the unweighted (Figs. 2A-2L) and weighted (Figs. 3A-3L) UniFrac-based food distances (*p>*0.05).

### Dietary diversity in associations with lifestyle factors and metabolic diseases

The association between the diversity of diet consumption and lifestyle factors and metabolic diseases is shown in (Additional File 1: eTable 2). All alpha-diversity indices of PUFA intake are significantly lower in hypertensive individuals than those without history of hypertension (Additional File 1: eFigures 6A-C). Both the distances for beta-diversity measurements showed the significant difference of diverse PUFA intake between smokers and non-smokers. However, the percentages of the variance explained by smoking status were observed to be very low (the R-square values of 1.95% and 1.88% for unweighted and weighted UniFrac distance metrics for PUFA intake diversity, respectively), which food source diversity of PUFA intake appeared not to be distinct (Additional File 1: eFigures 6D-E). The differences in within-subject dietary diversity of PUFA intake by history of diabetes and plant fat intake by history of hypertension were found; whereas between-subject dietary diversity of plant fat, carbohydrates, fiber, total fatty acids, and MUFA intake was associated with smoking status, depending on indices and measurements.

## Discussion

In this cohort of CRC patients, we investigated the microbiome variation according to different lifestyles, metabolic diseases, and diet consumption. While smokers, drinkers, and diabetic individuals had an increase in microbiome stochasticity (the AKP effect), the anti-AKP effects were presented in obese and hypertension individuals, compared to their counterparts.

The use of the AKP effects has been well established for the composition of microbiomes affected by external diverse stressors, such as predators, parasites, and social disruption, through the replacement or generation of locally deterministic changes of sensitive bacteria [6, 26]. Previous studies have demonstrated that modifiable factors have a strong effect on the structure and function of human gut microbial communities. Understanding these effects in a cohort of CRC patients is a vital goal in consulting recommendations through microbial ecology-based evidence. Here, we observed the AKP effects of smoking status, alcohol consumption, and diabetes, which indicates that smoking, drinking, and diabetes in CRC patients may cause a more variable and unstable microbiome structure due to the unavailability of the host to modulate their microbiome when disturbed. Consistently, findings from our network analyses indicated increased dispersion of bacteria in smokers, drinkers, and diabetic individuals than those of non-smokers, non-drinkers, and non-diabetics, respectively. In contrast, we observed a more stable microbiome composition among patients with elevated BMI or blood pressure compared to patients with low BMI or blood pressure, which indicated a more stable microbiome in obese or hypertensive patients than their counterparts.

Furthermore, there was greater dispersion of the microbiome composition in smoking and alcohol-consuming patients than in their counterparts, which was interpreted as dysbiosis with the less connected network in the dysbiotic group than in the non-dysbiotic group. Several biological mechanisms have been proposed that describe how numerous toxic chemicals in cigarette smoke, such as nicotine, aldehydes, and heavy metals, can affect the bacterial community through the peripheral immune system [27, 28]. Tobacco smoking has been shown to inhibit natural killer cell activities, enhance white blood cell counts, and increase infection susceptibility, which results in the impairment of antimicrobial defenses [27, 28]. Additionally, smoking can alter the gut microbiome by accumulating the gut taxon that promotes inflammation, such as *Bacteroides, Lachnospira, Prevotella stercorea*, and *Ruminococcus* [29]. Alcohol dependence has been associated with the onset of an inflammatory environment in the gut and alters the gut microbiome by deriving alcoholic metabolites and several neurotransmitters such as gamma-aminobutyric acid, serotonin, and dopamine [30–32].

The AKP effects of BMI remain controversial. Our study found a decrease in the gut microbiome variation related to obesity in CRC patients. However, Ma et al. observed a significantly higher similarity index in obese than lean individuals but a non-significant difference between overweight and lean subjects, indicating the presence of AKP effects in obesity only [7]. In general, a BMI in the overweight/obese range is related to the development of CRC through the mediators of systemic inflammation such as tumor necrosis factor-alpha and interleukin 6 [33, 34]. Inflammation also contributes to an increased risk of CRC via affecting obesity-related dysbiosis [35]. A meta-analysis of 1,301 participants revealed a lower microbiome diversity in obese compared to non-obese individuals without CRC, however, the diversity did not differ between obese and non-obese CRC patients [36], demonstrating the absence of AKP effects of obesity in individuals with CRC. Considering that Asian populations have a higher body fat percentage than non-Asian populations [37, 38], our study selected the cutoff BMI of 25 kg/m^2^ for obesity instead of the World Health Organization recommendation and observed the anti-AKP effect of obesity. We hypothesized that the different cutoffs of BMI affected our observation of AKP effects of obesity.

In this study, we found a decrease in the gut microbiome variation related to a history of hypertension but an increased heterogeneity related to underlying diabetes among CRC patients. Such bidirectional effects of the gut microbiome on blood pressure and fasting glucose level have been proposed [39, 40]. The overgrowth of the genus *Prevotella* and *Klebsiella* was shown to contribute to pre-hypertension and hypertension, and a large sample of Finns reported a weak association between an increase in several genera in the phylum *Firmicutes* and a decrease in many distinct *Lactobacillus* species in patients with blood pressure [41, 42]. The modulation of metformin, as well as other anti-diabetic agents to the microbial community, also received much-deserved interest [43, 44]. Nevertheless, the AKP effects of hypertension and type 2 diabetes have not been investigated before [45].

The concept of the AKP effect was introduced with the more extensive stochasticity and heterogeneity of microbiome composition between individuals, thus, leaded to a decrease in the ability of the immune responding to the exposure [6, 7]. However, the anti-AKP effect was further argued as an extreme case of the AKP effect [6]. Under mild conditions, dysbiosis in some individuals resulted in the difference of their microbial composition and increased between-individual variations [45]. Under severe conditions, dysbiosis occurred in most of individuals, which drastically reduced their microbial communities and made the microbial composition to be more similar between individuals [15].

Associations of food groups and dietary patterns with microbiome composition and diversity have been identified in the Asian population [46–48]. However, how the overall diet shapes the microbiome profile remains unclear. By constructing the tree-based food from the SQFFQ of 106 food items, we observed that diversity in terms of weight, energy, macronutrients, and fatty acid intake did not shape the gut microbial community in our cohort of CRC patients, which might be similar to the result of diets accounting for the only small proportion of microbiome variation in population-level studies [9, 49–51]. Notably, a recent ultra-dense longitudinal study revealed a high interaction of diet and microbiome at the individual level [45], which may explain our non-significant findings.

The concept of dietary diversity has been introduced as an indicator of nutrient adequacy and overall diet quality [52]. Despite the disparity from different calculation methods, pooled results of 16 individual studies did not find any significant associations between dietary diversity score with both overweight/obesity and BMI, which was consistent with our findings [53]. In addition, inverse associations between dietary diversity and the history of hypertension may partially support the reduced stochasticity of the microbiome community among hypertensive individuals due to their reduced composition and quantities of foods. However, the link between dietary diversity and microbiome variation did not show any significant findings.

The strength of the current study includes the use of the hierarchical tree for food-based consumption, which could reduce the dimension of complex dietary intake information. Besides, this study contains some limitations. First, from the nature of observational studies, there could be measurement errors in the assessment of modifiable factors and metabolic diseases due to recall bias. However, the utilization of a validated SQFFQ might minimize this error. Second, more than half of our study participants did not provide information on dietary intake, which may limit us in detecting the diet-microbiome relationship. We assumed those with and without dietary data were comparable because they did not differ in terms of general characteristics. Last, our study was unable to expand the AKP theory in the identification of the diversity-stability association and the assessment of the balance between deterministic and stochastic forces due to the lack of longitudinal data [7].

## Conclusion

In summary, our findings suggested an immune dysregulation and a reduced ability of the host and its microbiome in regulating the community composition. History of smoking, alcohol consumption, and diabetes were shown to affect parts of individuals in shifting new microbial communities and exert higher levels of stochasticity, whereas obesity and history of hypertension appeared to affect majority of individuals and shifted to drastic reductions in microbial compositions. Non-significant associations between dietary choices and microbiome diversity suggested the only small proportion of microbiome variation can be explained by dietary intake at the population level. Understanding the contribution of modifiable factors to differentiations of the gut microbiome among individuals may provide insights into how the microbiome regulates the effects of these factors on the health outcomes of CRC patients.

## Supplementary Information

**Additional file 1:** **eTable 1. **Hierarchical classification of foods at different levels. **eTable 2. ***P*-values from the generalized linear model for associations of the dietary alpha-diversity (Chao1, Shannon, Simpson indices) and from the permutational of variance tests for associations of dietary beta-diversity (unweighted UniFrac and weighted UniFrac distances) with lifestyle factors and metabolic diseases. **eFigure 1. **(A) Linear discriminant analysis (LDA) effect size (LEfSe) analysis and (B) cladogram for abundant bacteria in smokers and non-smokers. Yellowish circles indicate taxon which are not enriched in either smoking or non-smoking group. The diameter of each circle is proportional to the relative abundance. **eFigure 2. **(A) Linear discriminant analysis (LDA) effect size (LEfSe) analysis and (B) cladogram for abundant bacteria in drinkers and non-drinkers. Yellowish circles indicate taxon which are not enriched in either drinking or non-drinking group. The diameter of each circle is proportional to the relative abundance. **eFigure 3.** (A) Linear discriminant analysis (LDA) effect size (LEfSe) analysis and (B) cladogram for abundant bacteria in obese and normal weight individuals. Yellowish circles indicate taxon which are not enriched in either obesity or normal weight group. The diameter of each circle is proportional to the relative abundance. **eFigure 4.** (A) Linear discriminant analysis (LDA) effect size (LEfSe) analysis and (B) cladogram for abundant bacteria in hypertensive and nonhypertensive individuals. Yellowish circles indicate taxon which are not enriched in either hypertensive or non-hypertensive group. The diameter of each circle is proportional to the relative abundance. **eFigure 5.** (A) Linear discriminant analysis (LDA) effect size (LEfSe) analysis and (B) cladogram for abundant bacteria in diabetic and non-diabetic individuals. Yellowish circles indicate taxon which are not enriched in either diabetes or non-diabetes group. The diameter of each circle is proportional to the relative abundance. **eFigure 6. **Within- and between-subject dietary diversity of polyunsaturated fatty acid (PUFA) intake according to history of hypertension and smoking status. Box plots show dietary diversity indices [(A) Chao1, (B) Shannon, (C) Simpson] of PUFA supply within hypertensive and non-hypertensive individuals. Principal coordinate analysis plots based on (D) Unweighted UniFrac and (E) Weighted UniFrac show variation in food composition of PUFA supply between smoking and non-smoking individuals.

## Data Availability

The sequencing dataset supporting the conclusions of this article is available from the NCBI Sequence Read Archive (SRA) repository, [PRJNA797640, in https://www.ncbi.nlm.nih.gov/sra/PRJNA797640]. All other data are available from the corresponding author by reasonable request.

## Abbreviations

CRC: Colorectal cancer
SCFA: Short-chain fatty acid
AKP: Anna Karenina principle
BMI: Body mass index
AJCC: American Joint Committee on Cancer
SQFFQ: Semi-quantitative food frequency questionnaire
CAN-Pro: Computer-Aided Nutritional Analysis Program
COZINE: COpositional Zero-Inflated Network Estimation
LEfSe: Linear discrimination analysis effect size
MUFA: Monounsaturated fatty acid
PERMANOVA: Permutational multivariate analysis
PCR: Polymerase chain reaction
PUFA: Polyunsaturated fatty acid
