# Correction to: Dysbiotic microbiome variation in colorectal cancer patients is linked to lifestyles and metabolic diseases

**DOI:** 10.1186/s12866-023-02816-x

**Published:** 2023-03-15

**Authors:** Tung Hoang, Minjung Kim, Ji Won Park, Seung‑Yong Jeong, Jeeyoo Lee, Aesun Shin

**Affiliations:** 1 Department of Preventive Medicine, Seoul National University College of Medicine, 03080 Seoul, South Korea; 2 Integrated Major in Innovative Medical Science, Seoul National University College of Medicine, 03080 Seoul, South Korea; 3 Department of Surgery, Seoul National University College of Medicine, 03080 Seoul, South Korea; 4 Cancer Research Institute, Seoul National University, 03080 Seoul, South Korea

**Correction to:** ***BMC Microbiol*** **23** **, 33 (2023)**

10.1186/s12866-023-02771-7

The original version of this article [1] omitted Aesun Shin from the corresponding author list. As such, Minjung Kim and Aesun Shin have been denoted as co-corresponding authors.
