# Supplementary material for: Dysbiotic microbiome variation in colorectal cancer patients is linked to lifestyles and metabolic diseases
Source: BMC Microbiol. 2023 Jan 28;23:33. doi: 10.1186/s12866-023-02771-7 (PMC9883847; doi:10.1186/s12866-023-02771-7)
Supplement: Supplementary file 1 — Additional file 1: eTable 1. Hierarchical classification of foods at different levels. eTable 2. P-values from the generalized linear model for associations of the dietary alpha-diversity (Chao1, Shannon, Simpson indices) and from the permutational of variance tests for associations of dietary beta-diversity (unweighted UniFrac and weighted UniFrac distances) with lifestyle factors and metabolic diseases. eFigure 1. (A) Linear discriminant analysis (LDA) effect size (LEfSe) analysis and (B) cladogram for abundant bacteria in smokers and non-smokers. Yellowish circles indicate taxon which are not enriched in either smoking or non-smoking group. The diameter of each circle is proportional to the relative abundance. eFigure 2. (A) Linear discriminant analysis (LDA) effect size (LEfSe) analysis and (B) cladogram for abundant bacteria in drinkers and non-drinkers. Yellowish circles indicate taxon which are not enriched in either drinking or non-drinking group. The diameter of each circle is proportional to the relative abundance. eFigure 3. (A) Linear discriminant analysis (LDA) effect size (LEfSe) analysis and (B) cladogram for abundant bacteria in obese and normal weight individuals. Yellowish circles indicate taxon which are not enriched in either obesity or normal weight group. The diameter of each circle is proportional to the relative abundance. eFigure 4. (A) Linear discriminant analysis (LDA) effect size (LEfSe) analysis and (B) cladogram for abundant bacteria in hypertensive and nonhypertensive individuals. Yellowish circles indicate taxon which are not enriched in either hypertensive or non-hypertensive group. The diameter of each circle is proportional to the relative abundance. eFigure 5. (A) Linear discriminant analysis (LDA) effect size (LEfSe) analysis and (B) cladogram for abundant bacteria in diabetic and non-diabetic individuals. Yellowish circles indicate taxon which are not enriched in either diabetes or non-diabetes group. The diameter of each circle [file 12866_2023_2771_MOESM1_ESM.docx]

Dysbiotic microbiome variation in colorectal cancer patients is linked to lifestyles and metabolic diseases

Tung Hoang^1,2^, Min Jung Kim^3,*^, Ji Won Park^3^, Seung-Yong Jeong^3^, Jeeyoo Lee^1^, Aesun Shin^1,2,4,*^

^1^ Department of Preventive Medicine, Seoul National University College of Medicine, Seoul 03080, South Korea

^2^ Integrated Major in Innovative Medical Science, Seoul National University College of Medicine, Seoul 03080, South Korea

^3^ Department of Surgery, Seoul National University College of Medicine, Seoul 03080, South Korea

^4^ Cancer Research Institute, Seoul National University, Seoul 03080, South Korea

***Corresponding author details:**

Min Jung Kim, MD, PhD. Department of Surgery, Seoul National University College of Medicine, Seoul 03080, South Korea. Tel: +82-2-2072-7211. E-mail address: [minjungkim@snuh.org](mailto:minjungkim@snuh.org)

Aesun Shin, MD, PhD. Department of Preventive Medicine, Seoul National University College of Medicine, Seoul 03080, South Korea. Tel: +82-2-740-8331. E-mail: [shinaesun@snu.ac.kr](mailto:shinaesun@snu.ac.kr)

**eTable 1.** Hierarchical classification of foods at different levels.

| **Level** | **Food items** |
| --- | --- |
| Level 1 | Plant-based foods, animal-based foods, beverages, and condiments |
| Level 2 | 16 items: Cereals and grains, potatoes and starches, sugars and sweets, legumes, seeds and nuts, vegetables, mushrooms, fruits, meat and poultry, eggs, fish and shellfish, seaweed, milks and dairy, oils and fats, beverages, seasonings, and others |
| Level 3 | 35 items: Refined grains, whole grains, tubers and roots, noodles, rice cakes, bread, cereals and snacks, pizza and hamburger, cakes and sweets, legumes, tofu and soy milk, nuts and seeds, red meat, meat by products, processed meat, poultry, fish, seafood products, other seafood, salted fermented seafood, seaweeds, eggs, milk, dairy products, fruits, fruit products, green and yellow vegetables, light-colored vegetables, pickled vegetables, kimchi, mushrooms, oil and fat, condiments and seasonings, carbonated beverages, coffee and tea |
| Level 4 | 106 items: Cooked rice, cooked rice with soy beans, cooked rice with other cereals, cooked rice and cooked rice with soy beans almolst same, cooked rice and cooked rice with other cereals almost same, ramen, wheat noodles with soup, chajangmyeon/ jambbong, buckwheat vermicelli/ buckwheat noodle, dumping/dumping with soup, rice cake (plain rod shape)/ rice cake with soup, rice cake, cereals and corn flakes, loaf bread/ sandwich/ toast, jam/ honey/ butter/ margarine(when with put on bread), bread with small red bean, other bread, cakes/ chocopie, pizza/ hamburger, parched cereal powder, cookie/ cracker/ snack, candy/ chocolate, nuts, legumes (including beans, excluding cooked rice with bean), soup and stew with soybean paste/ soybean paste, eggs/ quail eggs, tofu (including tofu in soups and stews), starch jelly, potato (including fried, steamed boiled potatoes), sweet potatoes, starch vermicelli, kimchi and Korean cabbage, kkakduki/ small radish kimchi, kimchi and radish with water, other kimchi, Korean style pickles, radish/ salted radish, Korean cabbages/ Korean cabbage soup, spinach, lettuce, perilla leaf, vegetable wrap/ vegetable salad, other green vegetables, deokduck/ doraji, bean sprouts, bracken/ sweet potato stalk/ stem of taro, oyster mushroom, other mushrooms, pepper leaves/ chamnamul/ asterscaber, crown daisy/ leek/ water dropwort, cucumber, carot/ carrot juice, onion, green pepper, immature pumpkin, mature pumpkin/ pumpkin juice, pork (belly), roasted pork, braised pork, ham/ sausage, edible viscera, steak/ roasted beef, dog meat, fried chicken/ chicken stew, beef soup, beef soup with vegetables, sushi, mackerel/ Pacific saury/ Spanish mackerel, hair tail, eel, yellow croaker/ sea bream/ flat fish, Alaska Pollack, cuttlefish/ octopus, dried anchovy, canned tuna, salted-fermented fish, clam/whelk, oyster, crab, shrimp, fish paste/ crab flavored, dried laver, kelp/ sea mustard, milk, yogurt, ice cream, cheese, soybean milk, coffee, coffee sugar, coffee cream, green tea, carbonated drinks, other drinks, strawberry, muskmelon/ melon, watermelon, peach/ plum, banana, hard/ dried persimmon, tangerine, pear/ pear juice, apple/ apple juice, orange/ orange juice, grape/ grape juice, and tomato/ cherry tomato/ tomato juice |
| Level 5 | 663 items (refer to CAN Pro’s instruction) |

**eTable 2.** P-values from the generalized linear model for associations of the dietary alpha-diversity (Chao1, Shannon, Simpson indices) and from the permutational of variance tests for associations of dietary beta-diversity (unweighted UniFrac and weighted UniFrac distances) with lifestyle factors and metabolic diseases.

| **Dietary factor** | **Diversity index** | **Smoking** | **Drinking** | **Obesity** | **Hypertension** | **Diabetes** |
| --- | --- | --- | --- | --- | --- | --- |
| Weight consumption | Chao1 | 0.091 | 0.848 | 0.993 | 0.980 | 0.097 |
|  | Shannon | 0.199 | 0.756 | 0.177 | 0.536 | 0.897 |
|  | Simpson | 0.248 | 0.522 | 0.213 | 0.333 | 0.426 |
|  | Unweighted UniFrac | 0.053 | 0.891 | 0.392 | 0.343 | 0.089 |
|  | Weighted UniFrac | 0.115 | 0.911 | 0.283 | 0.099 | 0.170 |
| Energy intake | Chao1 | 0.737 | 0.835 | 0.657 | 0.978 | 0.685 |
|  | Shannon | 0.203 | 1.00 | 0.212 | 0.368 | 0.529 |
|  | Simpson | 0.311 | 0.800 | 0.213 | 0.298 | 0.295 |
|  | Unweighted UniFrac | 0.071 | 0.789 | 0.492 | 0.244 | 0.089 |
|  | Weighted UniFrac | 0.103 | 0.896 | 0.278 | 0.093 | 0.130 |
| Plant protein | Chao1 | 0.383 | 0.936 | 0.625 | 0.895 | 0.194 |
|  | Shannon | 0.483 | 0.537 | 0.212 | 0.180 | 0.217 |
|  | Simpson | 0.469 | 0.302 | 0.176 | 0.181 | 0.186 |
|  | Unweighted UniFrac | 0.057 | 0.855 | 0.246 | 0.208 | 0.068 |
|  | Weighted UniFrac | 0.076 | 0.832 | 0.114 | 0.012 | 0.131 |
| Animal protein | Chao1 | 0.190 | 0.835 | 0.442 | 0.792 | 0.286 |
|  | Shannon | 0.405 | 0.962 | 0.692 | 0.996 | 0.447 |
|  | Simpson | 0.207 | 0.561 | 0.426 | 0.506 | 0.845 |
|  | Unweighted UniFrac | 0.324 | 0.568 | 0.831 | 0.654 | 0.378 |
|  | Weighted UniFrac | 0.703 | 0.644 | 0.154 | 0.264 | 0.433 |
| Plant fat | Chao1 | 0.995 | 0.587 | 0.992 | 0.929 | 0.488 |
|  | Shannon | 0.281 | 0.375 | 0.389 | **0.040** | 0.358 |
|  | Simpson | 0.357 | 0.352 | 0.335 | 0.035 | 0.392 |
|  | Unweighted UniFrac | **0.032** | 0.746 | 0.323 | 0.260 | 0.087 |
|  | Weighted UniFrac | 0.091 | 0.650 | 0.486 | 0.077 | 0.268 |
| Animal fat | Chao1 | 0.160 | 0.448 | 0.911 | 0.966 | 0.722 |
|  | Shannon | 0.776 | 0.831 | 0.926 | 0.992 | 0.667 |
|  | Simpson | 0.958 | 0.533 | 0.934 | 0.854 | 0.843 |
|  | Unweighted UniFrac | 0.113 | 0.332 | 0.881 | 0.736 | 0.308 |
|  | Weighted UniFrac | 0.578 | 0.761 | 0.236 | 0.181 | 0.349 |
| Carbohydrates | Chao1 | 0.167 | 0.294 | 0.456 | 0.939 | 0.698 |
|  | Shannon | 0.241 | 0.962 | 0.146 | 0.337 | 0.529 |
|  | Simpson | 0.364 | 0.924 | 0.128 | 0.340 | 0.368 |
|  | Unweighted UniFrac | 0.066 | 0.805 | 0.407 | 0.264 | 0.109 |
|  | Weighted UniFrac | **0.030** | 0.753 | 0.194 | 0.105 | 0.202 |
| Fiber | Chao1 | 0.608 | 0.894 | 0.279 | 0.266 | 0.987 |
|  | Shannon | 0.989 | 0.720 | 0.051 | 0.195 | 0.745 |
|  | Simpson | 0.992 | 0.353 | 0.074 | 0.174 | 0.925 |
|  | Unweighted UniFrac | **0.030** | 0.822 | 0.166 | 0.356 | 0.068 |
|  | Weighted UniFrac | 0.110 | 0.765 | 0.196 | **0.030** | 0.298 |
| Total fatty acids | Chao1 | 0.796 | 0.827 | 0.368 | 0.501 | 0.116 |
|  | Shannon | 0.349 | 0.519 | 0.338 | 0.270 | 0.163 |
|  | Simpson | 0.309 | 0.373 | 0.467 | 0.440 | 0.150 |
|  | Unweighted UniFrac | **0.031** | 0.515 | 0.478 | 0.248 | 0.050 |
|  | Weighted UniFrac | 0.559 | 0.834 | 0.166 | 0.477 | 0.692 |
| Saturated fatty acids | Chao1 | 0.462 | 0.917 | 0.437 | 0.521 | 0.455 |
|  | Shannon | 0.558 | 0.861 | 0.389 | 0.142 | 0.114 |
|  | Simpson | 0.469 | 0.870 | 0.86 | 0.861 | 0.092 |
|  | Unweighted UniFrac | 0.068 | 0.693 | 0.538 | 0.350 | 0.049 |
|  | Weighted UniFrac | 0.688 | 0.899 | 0.164 | 0.176 | 0.792 |
| Monounsaturated fatty acids | Chao1 | 0.629 | 0.656 | 0.637 | 0.289 | 0.155 |
|  | Shannon | 0.646 | 0.661 | 0.938 | 0.075 | 0.128 |
|  | Simpson | 0.484 | 0.196 | 0.269 | 0.355 | 0.421 |
|  | Unweighted UniFrac | **0.014** | 0.328 | 0.56 | 0.523 | 0.111 |
|  | Weighted UniFrac | 0.653 | 0.764 | 0.572 | 0.053 | 0.223 |
| Polyunsaturated fatty acids | Chao1 | 0.221 | 0.549 | 0.141 | **0.042** | 0.053 |
|  | Shannon | 0.143 | 0.066 | 0.233 | **0.041** | **0.017** |
|  | Simpson | 0.166 | 0.116 | 0.160 | **0.039** | **0.006** |
|  | Unweighted UniFrac | **0.020** | 0.460 | 0.517 | 0.224 | 0.057 |
|  | Weighted UniFrac | **0.043** | 0.383 | 0.435 | 0.056 | 0.201 |

Bold font indicates significant associations.

**eFigure 1. (A)** Linear discriminant analysis (LDA) effect size (LEfSe) analysis and **(B)** cladogram for abundant bacteria in smokers and non-smokers. Yellowish circles indicate taxon which are not enriched in either smoking or non-smoking group. The diameter of each circle is proportional to the relative abundance.

**eFigure 2. (A)** Linear discriminant analysis (LDA) effect size (LEfSe) analysis and **(B)** cladogram for abundant bacteria in drinkers and non-drinkers. Yellowish circles indicate taxon which are not enriched in either drinking or non-drinking group. The diameter of each circle is proportional to the relative abundance.

**eFigure 3. (A)** Linear discriminant analysis (LDA) effect size (LEfSe) analysis and **(B)** cladogram for abundant bacteria in obese and normal weight individuals. Yellowish circles indicate taxon which are not enriched in either obesity or normal weight group. The diameter of each circle is proportional to the relative abundance.

**eFigure 4. (A)** Linear discriminant analysis (LDA) effect size (LEfSe) analysis and **(B)** cladogram for abundant bacteria in hypertensive and non-hypertensive individuals. Yellowish circles indicate taxon which are not enriched in either hypertensive or nonhypertensive group. The diameter of each circle is proportional to the relative abundance.

**eFigure 5. (A)** Linear discriminant analysis (LDA) effect size (LEfSe) analysis and **(B)** cladogram for abundant bacteria in diabetic and non-diabetic individuals. Yellowish circles indicate taxon which are not enriched in either diabetes or non-diabetes group. The diameter of each circle is proportional to the relative abundance.

**eFigure 6.** Within- and between-subject dietary diversity of polyunsaturated fatty acid (PUFA) intake according to history of hypertension and smoking status. Box plots show dietary diversity indices [**(A)** Chao1, **(B)** Shannon, **(C)** Simpson] of PUFA supply within hypertensive and non-hypertensive individuals. Principal coordinate analysis plots based on **(D)** Unweighted UniFrac and **(E)** Weighted UniFrac show variation in food composition of PUFA supply between smoking and non-smoking individuals.
